# Association between suicidal ideation and tandem repeats in *contactins*

**DOI:** 10.3389/fpsyt.2023.1236540

**Published:** 2024-01-04

**Authors:** Kairavi Parikh, Andrea Quintero Reis, Frank R. Wendt

**Affiliations:** ^1^Forensic Science Program, University of Toronto, Mississauga, ON, Canada; ^2^Biostatistics Division, Dalla Lana School of Public Health, University of Toronto, Toronto, ON, Canada; ^3^Department of Anthropology, University of Toronto, Mississauga, ON, Canada

**Keywords:** suicide, genetics, tandem repeats, *contactins*, mental health, adolescent psychiatry

## Abstract

**Background:**

Death by suicide is one of the leading causes of death among adolescents. Genome-wide association studies (GWAS) have identified loci that associate with suicidal ideation and related behaviours. One such group of loci are the six *contactin* genes (*CNTN1-6*) that are critical to neurodevelopment through regulating neurite structure. Because single nucleotide polymorphisms (SNPs) detected by GWAS often map to non-coding intergenic regions, we investigated whether repetitive variants in *CNTN*s associated with suicidality in a young cohort aged 8 to 21. Understanding the genetic liability of suicidal thought and behavior in this age group will promote early intervention and treatment.

**Methods:**

Genotypic and phenotypic data were obtained from the Philadelphia Neurodevelopment Cohort (PNC). Across six *CNTN*s, 232 short tandem repeats (STRs) were analyzed in up to 4,595 individuals of European ancestry who expressed current, previous, or no suicidal ideation. STRs were imputed into SNP arrays using a phased SNP-STR haplotype reference panel from the 1000 Genomes Project. We tested several additive and interactive models of locus-level burden (i.e., sum of STR alleles) with respect to suicidal ideation. Additive models included sex, birth year, developmental stage (“DevStage”), and the first 10 principal components of ancestry as covariates; interactive models assessed the effect of STR-by-DevStage considering all other covariates.

**Results:**

*CNTN1*-[T]_N_ interacted with DevStage to increase risk for current suicidal ideation (*CNTN1*-[T]_N_-by-DevStage; *p* = 0.00035). Compared to the youngest age group, the middle (OR = 1.80, *p* = 0.0514) and oldest (OR = 3.82, *p* = 0.0002) participant groups had significantly higher odds of suicidal ideation as their STR length expanded; this result was independent of polygenic scores for suicidal ideation.

**Discussion:**

These findings highlight diversity in the genetic effects (i.e., SNP and STR) acting on suicidal thoughts and behavior and advance our understanding of suicidal ideation across childhood and adolescence.

## 1 Introduction

Suicide is among the leading causes of death worldwide for adolescents. In Canada, it is the second leading cause of death for individuals between the ages of 15–34, and ninth across all age groups (1). Similarly, in the United States of America, suicide is the second leading cause of death among individuals between the ages of 10 and 24 (2). For every death by suicide, there are an estimated 20–25 additional suicide attempts (3, 4). Not only does this take an emotional toll on society but it also presents a hefty economic and public health burden. Friends and families of affected individuals are impacted psychologically and financially by the high costs associated with hospital visits, treatments, and often, bereavement leave from work (5). Death by suicide is not itself a psychiatric disorder; however, there is a strong correlation between diagnosis of a psychiatric disorder and suicidal behavior. For example, depression symptoms are often cited as one strong contributor to suicidal behavior (6). Adoption, twin, and family studies also consistently report a heritable component to suicidal thought and behavior. For example, in 2000, Powell and colleagues discovered that a family history of death by suicide was 4.6 times more prevalent in psychiatric inpatients who died by suicide than those who did not, even after adjusting for situational risk factors (7). Despite numerous studies indicating a heritable component to suicide traits, there is limited understanding of specific genetic associations to these traits in young individuals.

Genome-wide association studies (GWAS) play a crucial role in suicide research by comprehensively scanning the entire human genome to identify single nucleotide polymorphisms (SNPs) associated with a specific trait. In 2023, the largest GWAS on suicide attempt was performed by Docherty et al. (8). They discovered 12 genome-wide significant associations and genome-wide overlap with attention deficit hyperactivity disorder, smoking, and risk tolerance (8). This supported previous literature in that suicidal behavior is strongly correlated with psychiatric disorders and related psychopathologies. In the study, they found that after accounting for the effects of depression and posttraumatic stress disorder within a GWAS of suicide attempt, its overlap with other psychiatric disorders persisted, thus, showcasing the importance of identifying genetic variants unique to suicidal thoughts and behaviors (3). In the past, studies have found it challenging to isolate the genes responsible for suicidal behavior from those overlapping with other psychiatric disorders. The polygenic nature of suicide has also presented an obstacle in identifying suicide-specific genetic risks (6). Several GWAS of suicidal thought and behavior have been conducted and reveal a growing polygenic contribution that is independent of psychiatric diagnoses (3, 8). GWAS have also been conducted on suicidality by aggregating a range of discrete thoughts and behaviors related to a broad suicide severity spectrum. Notably, several significant studies have identified a correlation between the *contactin* (*CNTN*) gene family and suicidality (9, 10). Genetic variation in and around *CNTNs* have the potential to disturb neurodevelopmental functions, resulting in behavioral dysfunction such as depression and suicidal behavior. CNTNs are strong candidates for suicide-related association studies as they encode a protein called glycosylphosphatidylinositol. This protein is part of an immunoglobulin superfamily that is almost exclusively expressed in the central nervous system and plays an essential role in the formation of neural connections (8, 11). There are six members in the CNTN immunoglobulin family: CNTN1, CNTN2, CNTN3, CNTN4, CNTN5, and CNTN6. CNTN1 and CNTN2 are involved in axon myelination, neurite outgrowth, and neural cell adhesion and migration (12). CNTN3, CNTN4, CNTN5, and CNTN6 are involved in neurite branching, elongation, and neuronal connections (13). Disturbance to these neurodevelopmental functions is associated with the onset of multiple neurological disorders (14). Understanding genetic associations between *CNTNs* and suicide outcomes aids in advancing our understanding of the genetic liability for mental health and its related psychopathologies.

While numerous SNPs have been detected by GWAS of suicidal thoughts and behavior, including those in the *CNTN* family, they often localize to intergenic regions, complicating the translation of GWAS-based gene discovery to relevant biological mechanisms or processes leading to suicidal behavior. We hypothesized that studying variants with greater localization to coding and/or regulatory regions (e.g., introns, exons, and untranslated regions) of the genome can facilitate discovery of biology relevant for suicidal ideation. One such locus type is tandem repetitive elements (TREs). TREs are repetitive DNA motifs that may expand or contract at appreciable frequencies in the general population. Because of this behavior, TREs may be multi-allelic and offer greater resolution of the genotype–phenotype relationship by linking the length of the TRE to gene expression and trait variation (15–20). Extensive research has connected TRE variation to rare disorders like Huntington’s disease, muscular dystrophies, and some neurological conditions (21). However, more recent work has demonstrated a large and independent contribution of TRE variation to common complex traits like height, cholesterol concentration, and blood traits (16, 17, 19). The purpose of this study was to determine if there is an association between TREs in CNTNs and suicidal ideation in adolescents between the ages of 8 and 21. We focus on the “short” variety of TREs called short tandem repeats (STRs) that have repeat motifs ranging in size from 1 to 6 basepairs. It was recently shown that select STR genotypes can be imputed from genome-wide SNP arrays with relatively high accuracy (22). Using this imputation pipeline and subsequent association testing, we report additive and interactive associations between STR variations in CNTNs that increase risk for suicidal ideation in young people. This work provides further support for a genetic basis to suicidal thought and behavior, and contextualizes known genetic findings of suicide phenotypes with respect to study participant age. We expect these insights regarding genetic predispositions to suicidal ideation to benefit society by facilitating early intervention and treatment of suicidal behavior in youth.

## 2 Materials and methods

### 2.1 Data description

Genotypic and phenotypic data were obtained from the United States National Institute of Health’s (NIH) public repository, the database of genotypes and phenotypes (dbGaP). The data originate from the study “Neurodevelopmental Genomics: Trajectories of Complex Phenotypes” (dbGaP Study Accession ID: phs000607.v3.p2) (23). This study was performed in collaboration by the Center for Applied Genomics at Children’s Hospital of Philadelphia (CHOP) and the Brain Behavior Laboratory at the University of Pennsylvania in an effort to collect data for the Philadelphia Neurodevelopmental Cohort (PNC). Youth who visited CHOP for routine visits were asked to participate in genomic studies of complex pediatric conditions. The cohort consisted of 9,496 individuals, both male and female between the ages of 8 and 21 at the time of assessment. The cohort is considered generally healthy with no recruitment emphasis on any specific disorder, behavior, or trait. Clinical testing for each participant consisted of (i) screening via GOASSES (a modified version of the Kiddie-Schedule for Affective Disorders and Schizophrenia) to identify timeline of life events, demographics, medical history, and interviewer observations, (ii) a psychopathology symptom assessment of mood disorders (mania/hypomania), anxiety disorders, behavioral disorders, psychosis spectrum, eating disorders, suicidal thinking and behavior, and treatment history, and (iii) an abbreviated form of the Family Interview for Genetics Studies to assess major domains of psychopathology in the proband’s first-degree relatives (23). Participants were required to be proficient in English and be able to provide informed consent unless under the age of 18, for whom parental consent was obtained (23). Participants were excluded from the PNC if they had severe anxiety, medical disorders that could affect neuroimaging participation (e.g., brain tumors, head trauma, and blindness), and any conditions that could interfere with MRI scanning (e.g., metallic inserts, pregnancy). To determine if a participant had any of these exclusionary conditions, all participants were subject to clinical assessment, which consisted of a neuropsychiatric interview and a review of their electronic medical records.

Whole genome genotyping was performed using Affymetrix and Illumina SNP arrays. Pre-imputation quality control was performed according to the RICOPILLI pipeline^1^ (24). We first removed SNPs with a call rate < 0.95. We then applied a sample call rate of 0.98, removed related individuals with FHET +/− 0.2 prioritizing the retention of cases over controls, and excluded individuals whose genetic sex did not match their pedigree assignment. SNPs were then removed if they had a call rate < 0.98, missingness > 0.02, minor allele frequency < 0.05, and Hardy–Weinberg equilibrium value of *p* < 1 × 10^−6^. Participants were clustered using genetic data into one of the following global ancestry groups using a random forest classifier and a combined reference panel from the Human Genome Diversity Project plus the 1000 Genomes Project (Phase 3 unrelated participants only): Admixed American, African, Central/South Asian, East Asian, European, Oceanian, and Middle Eastern (25). Only participants with a European ancestry were analyzed in this study due to the limited availability of suicide phenotype data for the other ancestries and the greater STR imputation accuracy in this population group (22). A total of 4,595 participants were classified as consistent with predominantly European ancestry. To mitigate the effects of within-population genetic diversity, the first 10 principal components (PCs) were included as covariates for all statistical analyses.

Between 828 and 4,595 European ancestry participants answered suicide-related questions (see Table 1). Each question reflects a survey item originally described in the National Comorbidity Survey: Adolescent Supplement (NCS-A) (26, 27). The NCS-A was designed to (i) estimate the lifetime-to-date and current prevalence, age-of-onset distributions, course, and comorbidity of Diagnostic and Statistical Manual of Mental Disorders (DSM fourth Edition) disorders in the child and adolescent years of life among adolescents in the United States, (ii) to identify risk and protective factors for the onset and persistence of these disorders, (iii) to describe patterns and correlates of service use for these disorders, and (iv) to lay the groundwork for subsequent follow-up studies that can be used to identify early expressions of adult mental disorders. Out of the nine suicide phenotypes assessed, only three were applicable to this analysis as they (i) had at least 50 cases and (ii) had at least 200 total samples who responded to the question. These standards are commonly used for large genetic investigations (28). Based on this trait inclusion criteria, three suicidality phenotypes SUI001 (*N* = 4,595), SUI002 (*N* = 4,592), and SUI009 (*N* = 828) were included (Table 1). Responses for these three phenotypes reflect self-reported endorsement of the suicidal ideation item and were encoded as a 1 or a 0, where 1 indicates “yes” and 0 indicates “no.”

**Table 1:** Suicidality phenotypes in the PNC (dbGaP study accession phs000607.v3.p2).

Name	Question
SUI001*	Have you ever thought a lot about death or dying?
SUI002*	Have you ever thought about killing yourself?
SUI003	How old were you the first time you thought about suicide? (Age)
SUI004	How old were you the first time you thought about suicide? (Months)
SUI005	How old were you the first time you thought about suicide? (Date Year)
SUI006	How old were you the last time you thought about suicide? (Age)
SUI007	How old were you the last time you thought about suicide? (Months)
SUI008	How old were you the last time you thought about suicide? (Date Year)
SUI009*	Are you currently (within the past month) having thoughts about suicide?

Asterisks indicate a trait that was eligible for analysis. For source material describing each item, please reference to references (26, 27).

### 2.2 STR imputation

Short tandem repeat genotypes for the PNC participants were imputed using plink binary files for SNPs across the entire genome. SNP-STR imputation relied on a reference haplotype panel characterized in the 1000 Genomes Project (22). SNPs were phased using Beagle (v4.1), which performs statistical phasing by estimating haplotypes from genotype data for multi-allelic loci (22). Following this, the conform-gt program was used to match target SNP information to the reference SNP-STR data. In the PNC, we imputed 472 TREs across six contactins: *CNTN1*: 73, *CNTN2*: 7, *CNTN3*: 37, *CNTN4*: 91, *CNTN5*: 216, and *CNTN6*: 48. Quality control measures were applied for imputed TREs to ensure that only those loci with high imputation accuracy were included for phenotype association analysis. STRs retained for analysis had imputation allelic concordance >0.9 (Supplementary Table S1), had >2 alleles, and had Hardy–Weinberg equilibrium *p* values >0.0001. Imputation concordance per-STR was evaluated using data from Saini et al. (22). Briefly, Saini et al. (22) defined genotype concordance for a single individual as 0 if neither imputed allele matched a sequenced alleles, 0.5 if one but not both imputed alleles match the sequenced alleles, and 1 if both imputed alleles matched the sequenced alleles at a genotype. Per STR, the genotype concordance was the average across all samples in the 1000 Genomes Project and Simons Simplex Collection. We extracted imputation concordance values for our tested STRs from the Supplementary material of Saini et al. (22). The number of TREs varied among each CNTN (*CNTN1*: 36, *CNTN2*: 2, *CNTN3*: 18, *CNTN4*: 44, *CNTN5*: 109, and *CNTN6*: 23), totaling to 232 TREs across all six genes (Supplementary Table S1; Supplementary Figure S1). The mean concordance between imputed genotypes and whole-genome sequencing genotypes across the 232 STRs was 0.98 ± 0.02.

### 2.3 Statistical analyses

For each of the 232 STRs, STR allele lengths per locus (maternal and paternal) were summed to create a single score at a locus, termed a “length sum.” STR length-sums were calculated using the number of repeat units in a genotype and represented the locus-level burden of each STR (16, 17, 19). These scores were standardized to fit an approximate normal distribution. The baseline generalized linear models (GLM) were fit to the STR length sums and binary phenotype data using the binomial feature and included birth year, developmental stage (“DevStage”), biological sex, and the first 10 PCs of genetic ancestry. Multiple testing correction was applied per chromosome using the 5% false discovery rate method to account for the correlation between suicide phenotypes and STRs. In addition to the main effects of STRs tested using GLM, baseline interactive models included the interaction between the *CNTN* variants and participants’ DevStage in relation to suicidality phenotypes. Each model was subsequently tested using polygenic scores as additional covariates.

### 2.4 Polygenic scoring

Polygenic scoring (PGS) was applied to evaluate the independence of the STR additive and interactive effects on suicidal ideation. PGS were calculated using SNP effect sizes estimated from two GWAS of suicidal ideation and self-harm: “thought life was not worth living” (TLNWL, *N* = 88,456) and “thoughts of self-harm” (TSH, *N* = 82,959). Briefly, these GWAS were conducted in European ancestry participants of the United Kingdom Biobank using ordinal regression that included age, sex, genotyping chip, and the first eight PCs of ancestry as covariates (9). PGS were calculated using PRS-CS, a Bayesian polygenic prediction method, which infers posterior SNP effect sizes under continuous shrinkage priors (29). Incorporating shrinkage in PGS calculations enhances the generalizability and predictive performance of PGS in statistical modeling. LD-independent SNPs were selected based on the United Kingdom Biobank European ancestry reference panel. To test the interaction between SNPs and STRs, SNPs were excluded from PGS if they fell within 10 Mb surrounding the tandem repeat. The additive models included the full PGS for each suicide trait. Once calculated, PGS per PNC participant were included as additional covariates in the additive and interactive models. The interaction between TSH and TLNWL PGS with the baseline STR interactive models was also evaluated using GLM. Note that larger and more contemporary GWAS of suicide traits are available; however, we chose to calculate PGS from UKB GWAS to incorporate SNP effect sizes not confounded by the presence of military exposed participants which make up a large portion of other relevant suicide GWAS.

## 3 Results

### 3.1 Cohort features and trait distribution

The PNC data consisted of 9,496 participants from which a subset of individuals displaying suicidality phenotypes were identified. Three suicidality phenotypes in the PNC had suitable sample sizes for us to associate with imputed STR variation. These were SUI001 = 4,595, SUI002 = 4,592, and SUI009 = 828. These individuals endorsed either current, previous, or no suicidal ideation. The total number of phenotype cases for SUI001 and SUI002 were 682 and 364, respectively. The total number of SUI009 phenotype cases were 167 and the greatest number of SUI009 cases were observed in the middle proband (see Table 2). The young and adult probands consisted of nearly an equal number of cases but the ratio of cases: controls among the adult proband was higher (5.1%) than the young proband (2.8%) due to total sample size. The middle proband had a SUI009 case: control ratio of 3.6%. Overall, there was a higher representation of females in the PNC data, but this factor was accounted for during statistical analysis by the use of a biological sex covariate.

**Table 2:** PNC sample description by developmental stage.

Proband developmental stage	Age (years) at time of study participation	Male *N*	Female *N*	Total *N*	SUI001 Case *N*	SUI002 Case *N*	SUI009 Case *N*
Young	8–13	854	670	1,526	130	41	43
Middle	14–19	1,094	1,181	2,283	371	200	82
Adult	20+	364	464	829	181	123	42

**Figure 1:**
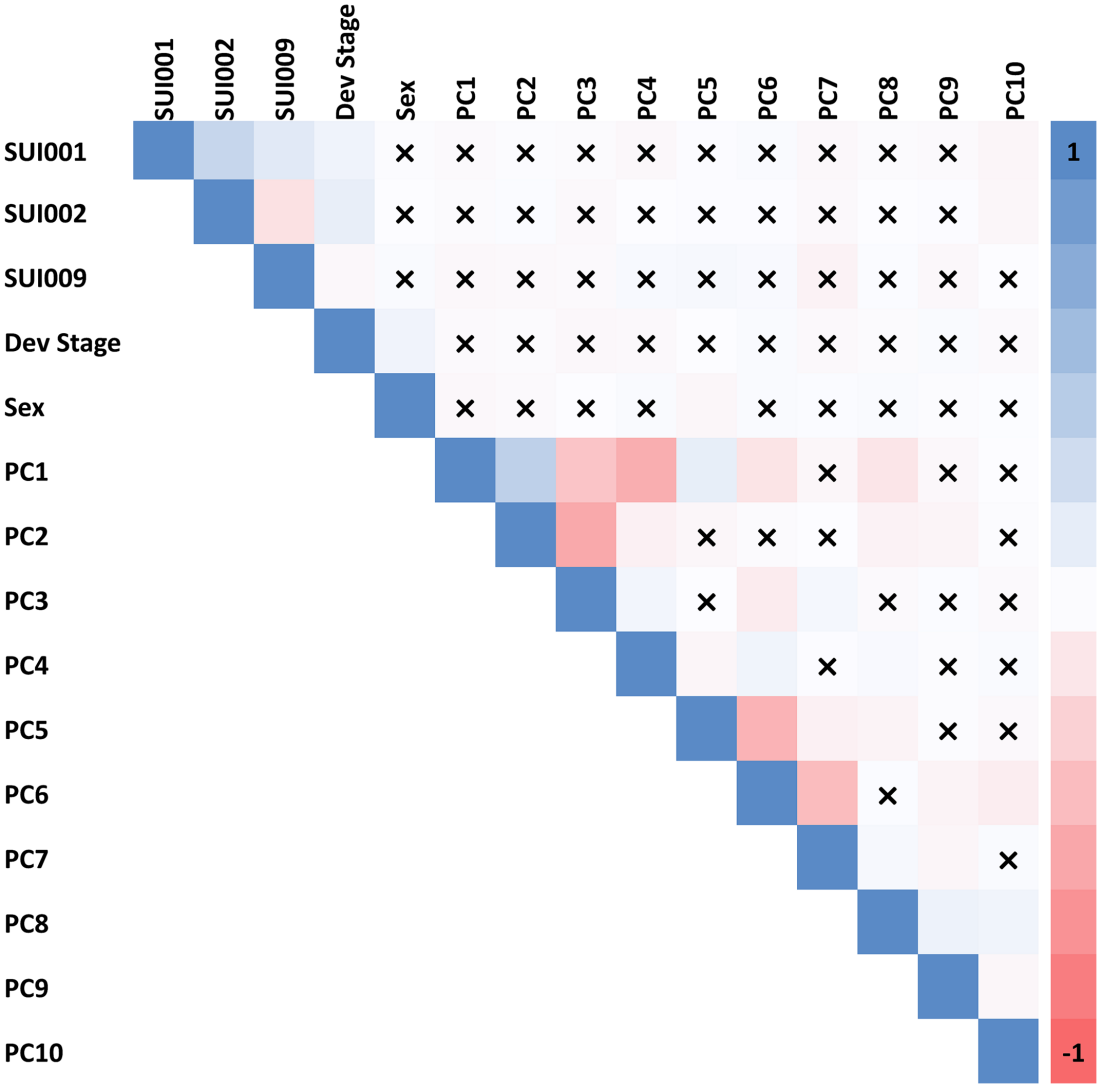
Spearman correlation among 12 covariates (developmental stage, biological sex, and 10 PCs of genetic ancestry) and phenotype variables. “*X*” indicates a correlation with non-significant *p* value (*p* > 0.05). SUI001 = “Have you ever thought a lot about death or dying?”; SUI002 = “Have you ever thought about killing yourself?”; and SUI009 = “Are you currently (within the past month) having thoughts about suicide/death/dying/killing yourself?”.

We first tested for correlation between each suicide outcome and commonly included covariates in genetic studies: birth year, developmental stages (captured by interview type), biological sex, and the first 10 principal components (PCs) of genetic ancestry (see Figure 1). A high correlation was observed between respective PCs but little to no correlation was observed between PCs and suicide phenotypes. Note that although the PNC suicide phenotypes were weakly correlated with genetic PCs, we opted to include PCs as covariates in the models of each suicide outcome to account for residual population stratification among our European ancestry participants that could artificially induce associations between STR length-sums and the suicide outcomes tested.

### 3.2 Additive and interactive model summaries

**Figure 2:**
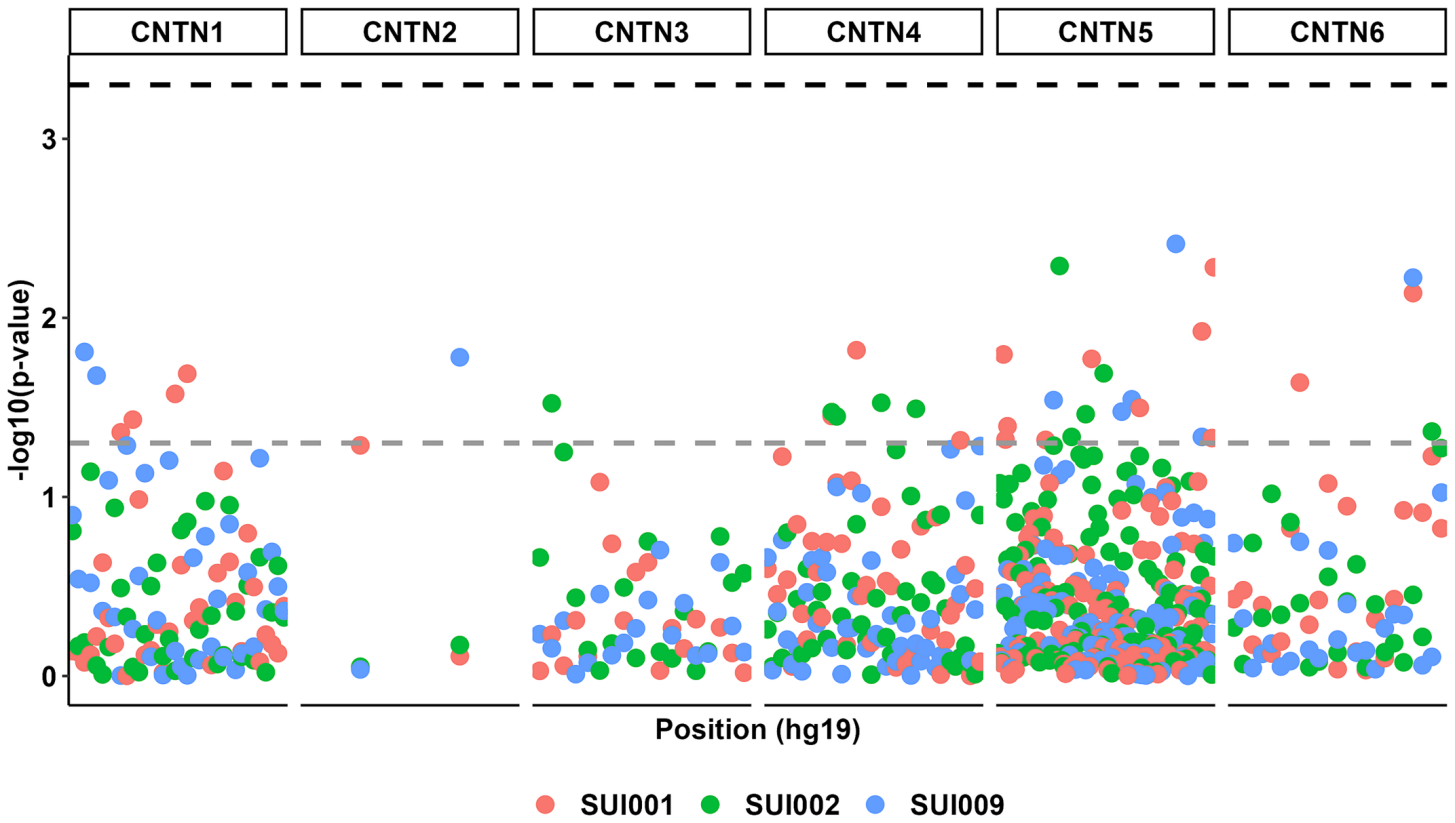
Manhattan plot for all genetic associations between *CNTN* family STRs (*n* = 232) and suicidal ideation phenotypes (*n* = 3). The gray dashed line represents a *p* value threshold of 0.05. The black dashed line represents a *p* value threshold of 0.0005 after considering a false discovery rate of 5%.

Despite multiple suggestive associations between *CNTN* STRs (*p* < 0.05), no additive model associations surpassed the corrected *p* value threshold (Figure 2; Supplementary Table S2).

**Figure 3:**
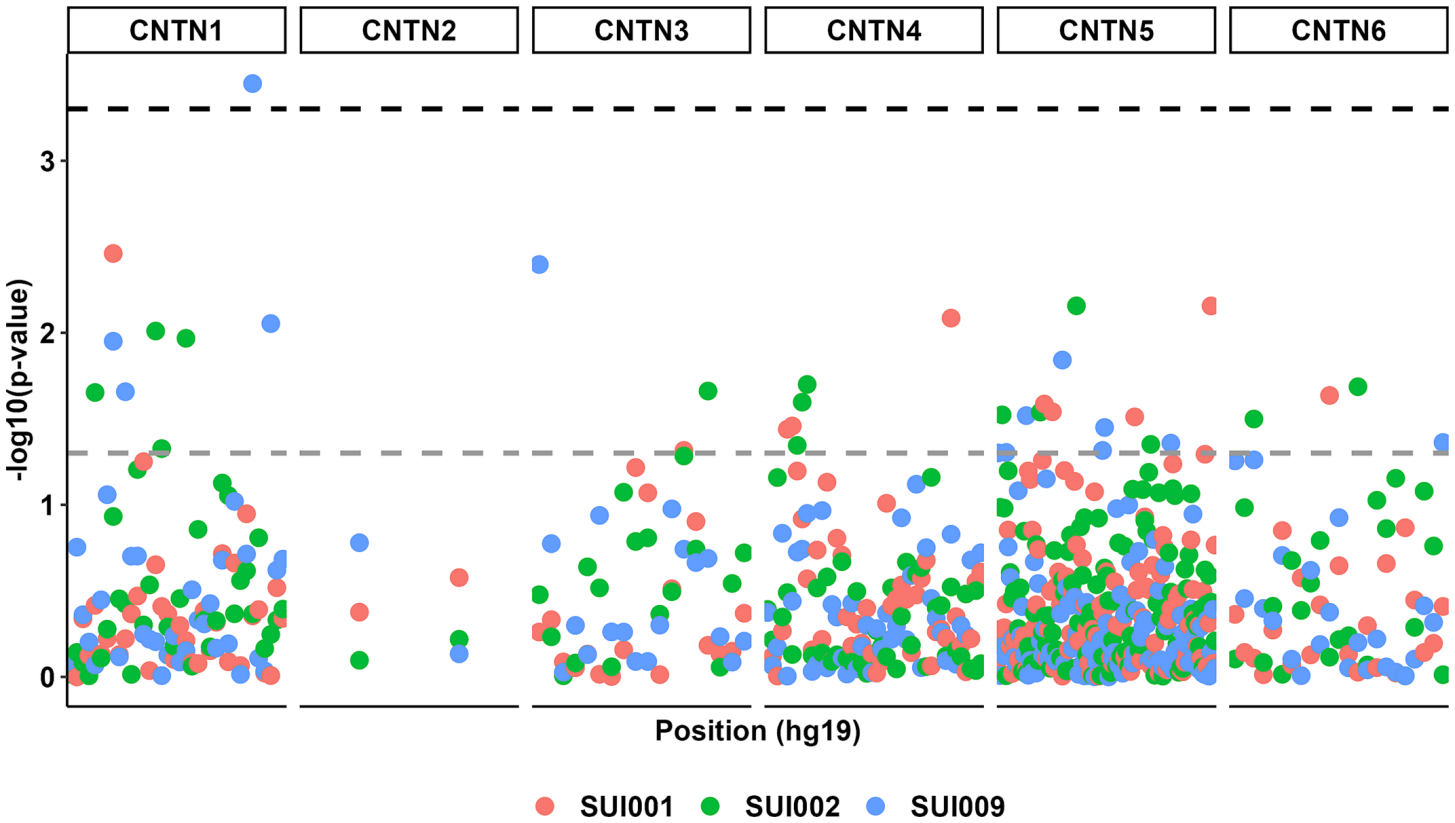
Manhattan plot for all genetic associations between *CNTN* family STRs (*n* = 232) and suicidal ideation phenotypes (*n* = 3) with respect to participant birth year. The gray dashed line represents a *p* value threshold of 0.05. The black dashed line represents a *p* value threshold of 0.0005 after considering a false discovery rate of 5%.

**Figure 4:**
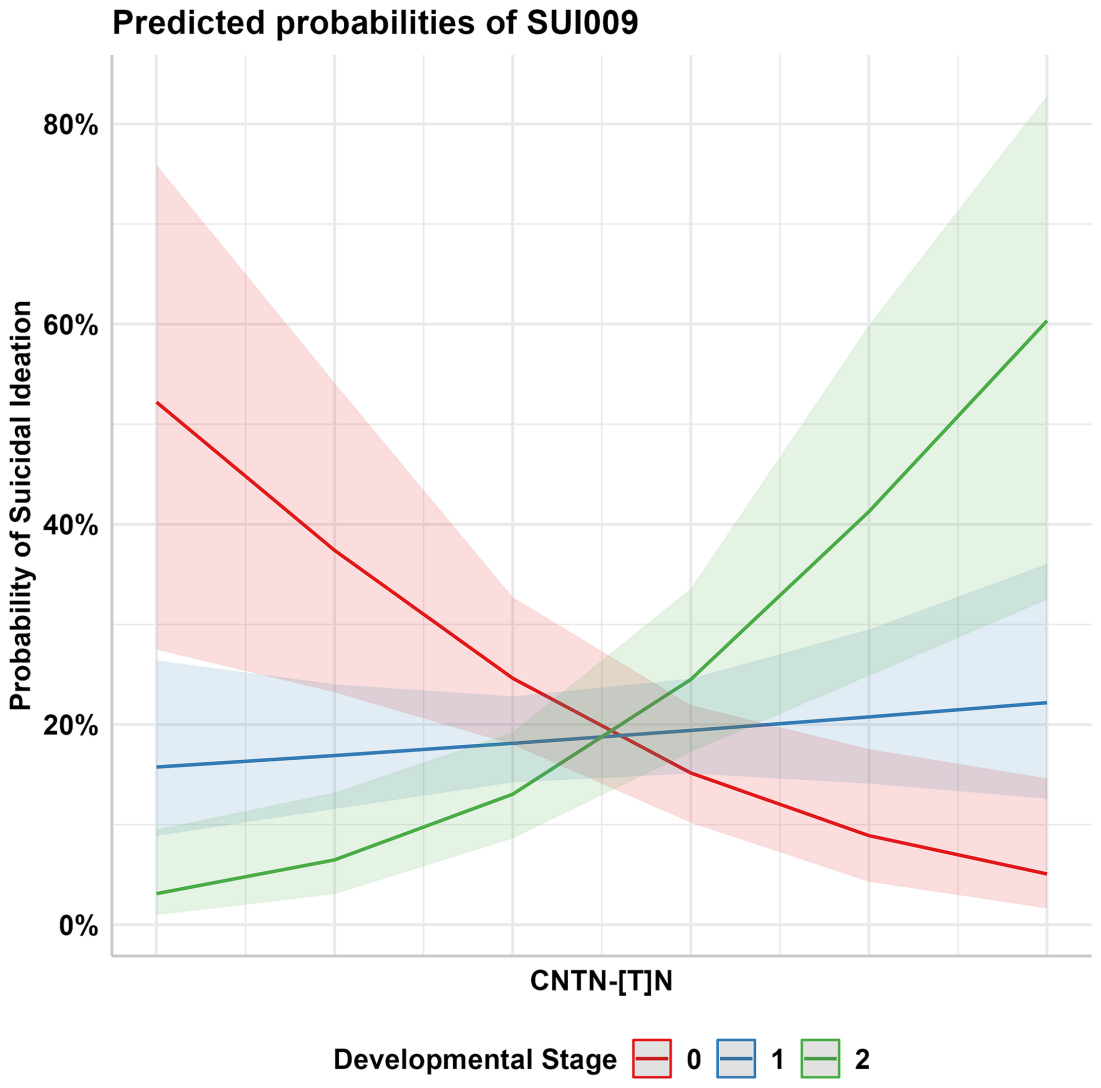
Probability of current suicidal ideation (PNC Trait ID SUI009) among participants aged 14–19 (Developmental Stage 1 OR = 1.80, SE = 1.35) and 20–21 (Developmental Stage 2 OR = 3.82, SE = 1.43) in comparison to the youngest participant group, aged 9–13 (Development Stage 0).

The birth year interactive GLM model between the 232 *CNTN* STRs and suicidality phenotypes resulted in multiple suggestive significant associations (*p* < 0.05, Figure 3); these associations were enriched for *CNTN1* STRs relative to the other genes (hypergeometric test, 1.59-fold enrichment, *p* = 0.049). After correcting for multiple testing (FDR < 5%), one STR interactive was significant. This association was observed between a *CNTN1* STR [STR ID: 295642, chr12:41415438 (hg19), and chr12:41021636 (hg38)] and the current suicidal ideation phenotype (SUI009; see Table 1), denoted by *CNTN1*-[T]_N_-by-DevStage (OR = 1.99, s.e. = 0.523, *p* = 3.56 × 10^−4^). This STR has a reference sequence of “TTTTTTTTTTTTTTTTTG” where the thymine homopolymer ranges from 15 to 20 repeats in the PNC data. As seen in Figure 4, with increasing developmental stage (i.e., decreasing birth year), there was an upward trend in suicidal ideation. The odds ratios for current suicidal ideation were significantly higher in the middle (OR = 1.80, *p* = 0.051) and adult (OR = 3.82, *p* = 2.00 × 10^−4^) participant groups. We considered the 1,391 common (minor allele frequency > 5%) SNPs within 500 kb of *CNTN1*-[T]_N_ to ensure the detected STR signal is independent of per-SNP effects in the surrounding area. In the same interactive model, no SNP was significantly associated with SUI009 after multiple testing correction (Supplementary Table S4; Supplementary Figure S2). The most significant finding was rs1602633-by-DevStage (OR = 1.15, s.e. = 0.360, *p* = 0.001, FDR = 0.222). There was no evidence that this SNP-by-development effect had an influence on the TR-by-development interaction. After conditioning, the *CNTN1*-[T]_N_-by-DevStage remained significant (OR = 1.91, s.e. = 0.542, *p* = 4.31 × 10^−4^).

We next evaluated whether the additive and interactive effects detected above were independent of genome wide PGS for suicidal ideation traits. Upon incorporating TSH and TLNWL PGS as additive covariates into the baseline *CNTN1*-[T]_N_ additive models (Table 2; Supplementary Table S5), there were no significant affects detected. Despite themselves being associated with suicidal ideation, the addition of TSH PGS and TLNWL PGS as additive covariates to the baseline interactive model demonstrated no statistically significant deviations from the baseline estimate (*p*_difference_ > 0.05). This suggests that they have little to no effect on the interaction detected between *CNTN1*-[T]_N_ and DevStage. Furthermore, incorporating a three-way interaction term where PGS, DevStage, and *CNTN1*-[T]_N_ variation contribute to suicidal ideation modestly increased the interaction between *CNTN1*-[T]_N_ and DevStage independent of all other covariates and permitted interactions among them. Across all models, the strongest effect was an interaction between *CNTN1*-[T]_N_ and DevStage (OR = 2.01, *p* = 2.17 × 10^−4^) that was independent of all additional interaction items including those with TSH PGS (Table 3).

**Table 3:** Statistical summary of all baseline additive and interactive STR (*CNTN1*-[T]_N_) models with polygenic scores (PGS) for suicidal thoughts and behaviors.

Model specifications	Effect (Odds ratio)	SE	*Z*-score	*p* value
Baseline additive^$^	1.052	2.499	0.421	0.674
Baseline additive^$^ + TSH PGS	1.049	2.619	0.402	0.688
Baseline additive^$^ + TLNWL PGS	1.054	2.401	0.439	0.661
Baseline interactive^#^	1.997	0.523	3.804	1.43×10^−4^
Baseline interactive^#^ + TSH PGS	1.965	0.523	3.759	1.70×10^−4^
Baseline interactive^#^ + TLNWL PGS	1.960	0.523	3.745	1.81×10^−4^
Baseline interactive^#,@^ -by-TSH PGS	2.014	0.544	3.700	2.17×10^−4^
Baseline interactive^#,@^ -by-TLNWL PGS	1.97	0.529	3.728	1.93×10^−4^

Each “additive” model treated the STR and developmental stage as independent items in the generalized linear model while each “interactive” model considered STR-by-DevStage effects. When PGS were added to the models, their interaction was with respect to STR-by-DevStage. TSH, “thoughts of self-harm”; TLNWL, “thought life was not worth living.” ^$^Baseline additive model: SUI009 ~ STR + DevStage + covariates. ^#^Baseline interactive model: SUI009 ~ STR*DevStage + covariates. ^@^Reported effect estimate corresponds to TR-by-DevStage in the presence of PGS as a third interacting variable; in other words, we report STR-by-DevStage, not STR-by-DevStage-by-PGS in this table. All GLM results are shown in Supplementary Table S5.

## 4 Discussion

As a leading cause of death among young people globally, we focused our study on investigating genetic factors associated with suicide outcomes in the PNC, a cohort of generally healthy young people. Appreciating that SNPs detected by GWAS are almost exclusively found in intergenic regions, we investigated a class of genetic variation that permits more fine-grained dose-dependent effects of allelic variation per locus in STRs. Current suicidal ideation [SUI009—Are you currently (within the past month) having thoughts about suicide?] was the only phenotype among the three suicidality phenotypes assessed in this study that displayed a statistically significant result with a *CNTN* variant. The interaction between locus *CNTN1*-[T]_N_ and the developmental stage of participants revealed that older age (20–21 years old) and longer homopolymeric stretches collectively have a 3.8-fold increase compared to a younger cohort with shorter homopolymeric stretches. The addition of TSH PGS and TLNWL PGS to the interactive models did not attenuate this signal.

CNTN1 is a neuronal cell adhesion molecule whose expression regulates neuro-immune function and axon growth in neurons (30). CNTN1 functions have been extensively studied in transgenic mice to understand the effects of high CNTN1 expression on neurological and inflammatory disorders. A recent study identified CNTN1 as a novel risk gene that induces anxiety and depression through functional actions in the hippocampus, which reduce neuronal growth and maturity (30). Given the vital role that CNTN1 plays in neuronal development processes, disruptions in the form of gene alterations (i.e., genetic variants or changes to gene expression levels) can significantly impact an individual’s mental growth and wellbeing. Since the *CNTN1* STR identified in this study is intronic, gene functionality can also be indirectly affected through repression of alternative splicing in primary mRNA transcripts, which alters the lengths of functional exons and consequently, affects protein structure (31). Despite efforts to identify whether the *CNTN1* STR found here has been previously implicated in gene expression (32) or alternative splicing (33), the locus in question was not assayed in previous reports. The finding in this study advances our understanding of the extent to which genetic variants in *CNTN1* can affect an individual’s mental health and lead to a state of suicidal ideation. Furthermore, with increasing age indicating a higher odds ratio for current suicidal ideation, it is worthwhile to acknowledge the effects of epigenetics on suicidality. It is well documented in literature that DNA methylation has a significant effect on depression and increased suicide risk (34). As one ages, they may accumulate and experience a variety of emotions, memories, and challenges. Environmental influences that gradually alter an individual’s methylation profile may affect their neuronal growth and activation by altering expression via methylation and other genetic variants, such as the STR detected here. Future studies should consider evaluating the presence of *CNTN1* variants in older populations to better understand the increasing odds of current suicidal ideation with age. Furthermore, investigations of how age-related methylation accumulation may interact with *CNTN1* STR variation should be considered as well.

The biology of CNTN1 is perhaps most well understood in the context of pain, a trait very highly correlated with suicidal behavior (8). CNTN1 has been proposed as a promising pain biomarker (35). Its interaction with neurofascin-155 plays an essential role in propagating pain signals and reduced CNTN1 activity/concentration results in chronic inflammatory demyelinating polyneuropathy and is considered a *bona fide* marker for pain. It is therefore possible that our detection of a *CNTN1* STR association with suicidal ideation, though not itself encoding CNTN1 protein structure, may be tagging mechanisms that link pain biology to suicidal psychopathology in young people (36). This link is supported by recent large-scale GWAS and warrants further targeted study (8).

## 5 Strengths and limitations

The strengths of this study are 3-fold. First, TR association testing is lacking in the common complex trait literature. Their study has the potential to identify powerful hypotheses linking genetic variation to trait or symptom severity (e.g., longer repeat lengths correlate with or cause more severe depressive symptoms). Second, despite young people being at incredibly high risk for death by suicide, youth and adolescent cohorts remain under investigated in terms of genetic factors associated with suicidal thought and behavior. We bridge this gap by explicitly modeling how developmental stage in a young cohort interacts with genetic data to explain variation in suicidal ideation. Our finding of a gene-by-age effect involving a family of immunoglobulins with clear influence on brain biology supports the explicit study of genetic underpinnings of mental health diagnoses and symptoms in young people. Furthermore, the detected interaction effect highlights the formative young adult years as a relevant timeframe for genetic risk for suicidal ideation to present itself. By helping young people develop, for example, strong social supports and a clear purpose in life prior to these formative years, one might hypothesize that these strong protective factors will mask genetic risk for suicidal ideation (37, 38). Finally, we demonstrate that the interactive effect uncovered is robust to genome-wide risk for suicidal thought and behavior, adding confidence to the reported effect.

It should be noted that this study has three main limitations and areas for improvement. First, the PNC evaluates broad aspects of human neurodevelopment with suicidal ideation being one feature of that assessment. We appreciate that suicidal ideation may fundamentally differ from acts like planning and attempt. Therefore, future studies should investigate a larger number of traits that reflect more complex behavior like suicidal actions and family history of suicidal behavior. Second, this study only considered the interaction between participant developmental stage and *CNTN* STR variants in relation to suicidality among European ancestry participants. Prior work supports unique environmental patterns that interact with genetic risk for suicide outcomes (39). Future work will be required to investigate how matrices of environments, including age, socioeconomic position, etc., interact with STR variation. Finally, our study is limited in its focus on European ancestry participants. Additional investigation into how diverse sociocultural experiences interact with the genetics of suicidal thought and behavior will provide essential information to advocate and support treatment of these thoughts across diverse communities.

## 6 Conclusion

We identified a novel genetic variant in *CNTN1* that is associated with current suicidal ideation in youth (*CNTN1*-[T]_N_-by-stage). We demonstrated that there is a positive relationship between the odds of current suicidal ideation and developmental stage with the presence of longer *CNTN1*-[T]_N_ variants. These findings provide valuable insights into the complex genetic underpinnings of suicidal ideation and underscore the importance of considering interactive effects of age and/or developmental stage in the planning of future research and prevention strategies.

## Data availability statement

The original contributions presented in the study are included in the article/Supplementary material; further inquiries can be directed to the corresponding author.

## Ethics statement

The studies involving humans were approved by University of Toronto Research Ethics Board (protocol 43714). The studies were conducted in accordance with the local legislation and institutional requirements. The human samples used in this study were acquired from an approved dbGaP study protocol. Written informed consent for participation was not required from the participants or the participants’ legal guardians/next of kin in accordance with the national legislation and institutional requirements.

## Author contributions

KP and FW: conceptualization. KP, AQ, and FW: formal analysis and writing—review and editing. FW: funding and supervision. KP: writing—original draft. All authors contributed to the article and approved the submitted version.

## Footnotes

^1^ https://sites.google.com/a/broadinstitute.org/ricopili/preimputation-qc
