# Supplementary material for: Association between suicidal ideation and tandem repeats in contactins
Source: Front Psychiatry. 2024 Jan 4;14:1236540. doi: 10.3389/fpsyt.2023.1236540 (PMC10794671; doi:10.3389/fpsyt.2023.1236540)
Supplement: Supplementary file 2 [file Data_Sheet_1.docx]

**Supplementary Material**


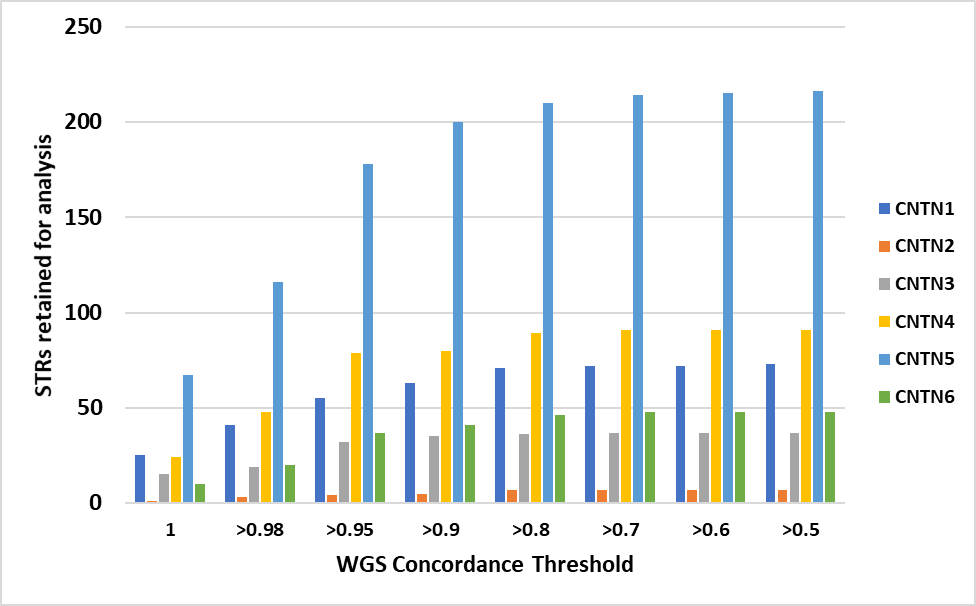


**Figure S1.** Distribution of concordance between imputed genotypes and actual genotypes in the 1000 Genomes Project and Simons Simplex Collection. Data reflect a subset of loci reported by Saini, et al. that encompass only the contactins (CNTNs) used in this study.


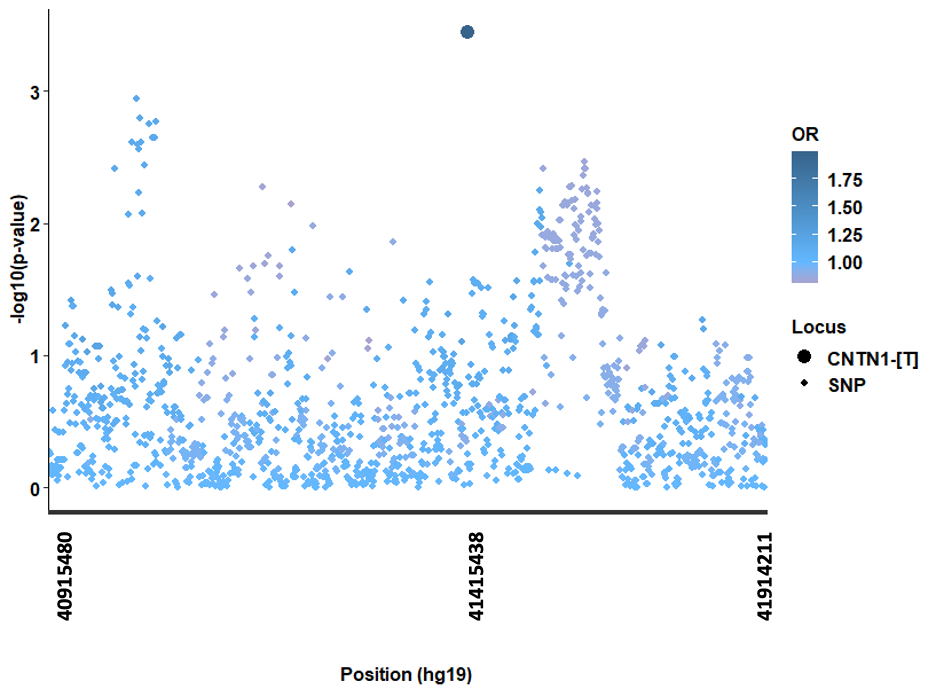


**Figure S2.** Local Manhattan plot for SUI009 (“*Are you currently (within the past month) having thoughts about suicide?”*) depicting the chromosome 12 region containing the STR *CNTN1*-[T]_N_ and all common (minor allele frequency >5%) SNPs within 500kb of the STR. Data points are colored by their odds ratio from an interaction model of SUI009 where each genetic variant was tested for an interaction with participant developmental stage (Table S4).
